# NHANES-based assessment of neutrophil-percentage-to-albumin and neutrophil-to-lymphocyte ratios as moderate predictors of mortality in adults with chronic respiratory diseases

**DOI:** 10.3389/fphar.2025.1582120

**Published:** 2025-09-26

**Authors:** Feng Xu, Pan Jiang

**Affiliations:** ^1^ Department of Intensive Care Unit, Shenzhen Guangming District People’s Hospital, Shenzhen, Guangdong, China; ^2^ Department of Stomatology, Shenzhen Guangming District People’s Hospital, Shenzhen, Guangdong, China

**Keywords:** neutrophil-percentage-to-albumin ratio, neutrophil-to-lymphocyte ratio, eosinophil-to-lymphocyte ratio, mortality, asthma, NHANES

## Abstract

**Objective:**

This study aimed to evaluate the predictive roles of hematologic inflammatory biomarkers, including the neutrophil-percentage-to-albumin ratio (NPAR), neutrophil-to-lymphocyte ratio (NLR), and eosinophil-to-lymphocyte ratio (ELR), in relation to mortality among individuals with chronic respiratory diseases (CRDs).

**Methods:**

We analyzed data from the National Health and Nutrition Examination Survey (NHANES) to assess the relationship between these inflammatory biomarkers and mortality in adults with CRDs. Multivariable Cox regression models were used to estimate hazard ratios (HRs) for all-cause mortality, adjusting for potential confounders. Receiver operating characteristic (ROC) curve analysis was employed to evaluate the predictive performance of these biomarkers, with the area under the curve (AUC) used to compare their accuracy.

**Results:**

A total of 8,387 participants with CRDs were included in the analysis. Higher levels of NPAR were significantly associated with increased all-cause mortality (HR = 1.33, 95% CI: 1.26–1.40, P < 0.001). Similarly, elevated NLR was associated with higher mortality risk (HR = 1.24, 95% CI: 1.17–1.30, P < 0.001). In contrast, ELR did not show a significant association with mortality (HR = 1.04, 95% CI: 0.99–1.10, P = 0.098). ROC curve analysis revealed that NPAR had the highest AUC value (0.639, 95% CI: 0.623–0.656), suggesting modest yet relatively better discriminative capacity among the evaluated biomarkers for mortality risk stratification.

**Conclusion:**

Among individuals with chronic respiratory diseases, higher NPAR and NLR are significant predictors of mortality, with statistically significant but moderate predictive ability as indicated by their AUC values. These findings suggest that NPAR and NLR may serve as useful biomarkers for risk stratification in patients with CRDs, though their clinical utility is limited by modest predictive power.

## Introduction

Chronic respiratory diseases (CRDs), including chronic bronchitis, emphysema and asthma, pose a significant global health challenge, affecting millions and contributing to high morbidity and mortality rates (Momtazmanesh et al., 2023). The World Health Organization (WHO) identifies CRDs as leading causes of death (Jung et al., 2022). The rising prevalence of these diseases is attributed to several factors, including aging populations, increased exposure to air pollution, and smoking (Adeloye et al., 2022). The economic burden associated with CRDs is substantial, encompassing healthcare costs, lost productivity, and disability. While these conditions share features of chronic inflammation, they exhibit distinct pathophysiological mechanisms: Asthma typically involves eosinophilic/Th2-mediated pathways, whereas COPD features neutrophilic inflammation and nutritional compromise. This heterogeneity complicates prognostic biomarker development.

Systemic inflammation is a hallmark of chronic respiratory diseases (CRDs) and is closely linked to disease progression and mortality (Rehman et al., 2021). This inflammation is characterized by an imbalance in immune responses, which can be quantified through various biomarkers. Recent studies have identified the neutrophil-percentage-to-albumin ratio (NPAR), neutrophil-to-lymphocyte ratio (NLR), and eosinophil-to-lymphocyte ratio (ELR) as potential indicators of systemic inflammation and disease outcomes in various conditions (Lessomo et al., 2023; Zinellu and Mangoni, 2024). Specifically, NPAR integrates the percentage of neutrophils in the total white blood cell count with serum albumin levels, reflecting both the inflammatory response and nutritional status (Yao et al., 2022). Elevated NPAR levels have been linked to increased mortality risk in various conditions, suggesting its utility as a prognostic marker in CRDs (Ma et al., 2024). The NLR, which measures the ratio of neutrophils to lymphocytes, provides insights into the balance between pro-inflammatory and anti-inflammatory responses, further elucidating the inflammatory milieu in CRDs (García-Escobar et al., 2023). NPAR integrates both the inflammatory response (reflected by neutrophil percentage) and nutritional status (indicated by serum albumin), providing a more holistic view of the patient’s systemic health than NLR or ELR alone. Elevated NPAR may thus reflect a more severe inflammatory burden coupled with underlying malnutrition or chronic disease state, both of which are known to be strong predictors of adverse outcomes.

Beyond CRDs, hematologic biomarkers like NLR and CRP/mean platelet volume ratio (CRP/MPV) demonstrate significant utility in stratifying disease severity across respiratory conditions. For instance, in community-acquired pneumonia (CAP), CRP/MPV and NLR effectively identify patients requiring hospitalization, enabling timely interventions that reduce morbidity and mortality (Yeşildağ et al., 2024). These findings underscore the broader applicability of accessible inflammatory markers in respiratory pathology.

The current study aims to fill this gap by conducting a comparative analysis of NPAR, NLR, and ELR to evaluate their predictive roles for all-cause mortality among adults with CRDs using data from the National Health and Nutrition Examination Survey (NHANES). We specifically hypothesize that NPAR will demonstrate superior predictive performance for mortality compared to NLR and ELR due to its ability to reflect both inflammatory response and nutritional status. The inclusion of ELR is critical to assess the role of eosinophilic inflammation and provide a more complete understanding of the diverse inflammatory mechanisms contributing to mortality in this heterogeneous patient group. Our findings may provide valuable insights for clinical practice, guiding the identification of high-risk patients and informing personalized treatment strategies.

## Methods

### Data source

Data for this study were obtained from NHANES, conducted by the Centers for Disease Control and Prevention (CDC) in the United States. NHANES is a nationally representative cross-sectional survey that assesses the health and nutritional status of the non-institutionalized civilian population of the United States. The survey employs a complex, stratified sampling design to ensure representativeness across various demographic groups, including age, sex, race, and socioeconomic status. This study utilized data from 11 survey cycles conducted between 2001 and 2018, providing a comprehensive dataset for analysis.

### Study population

**FIGURE 1:**
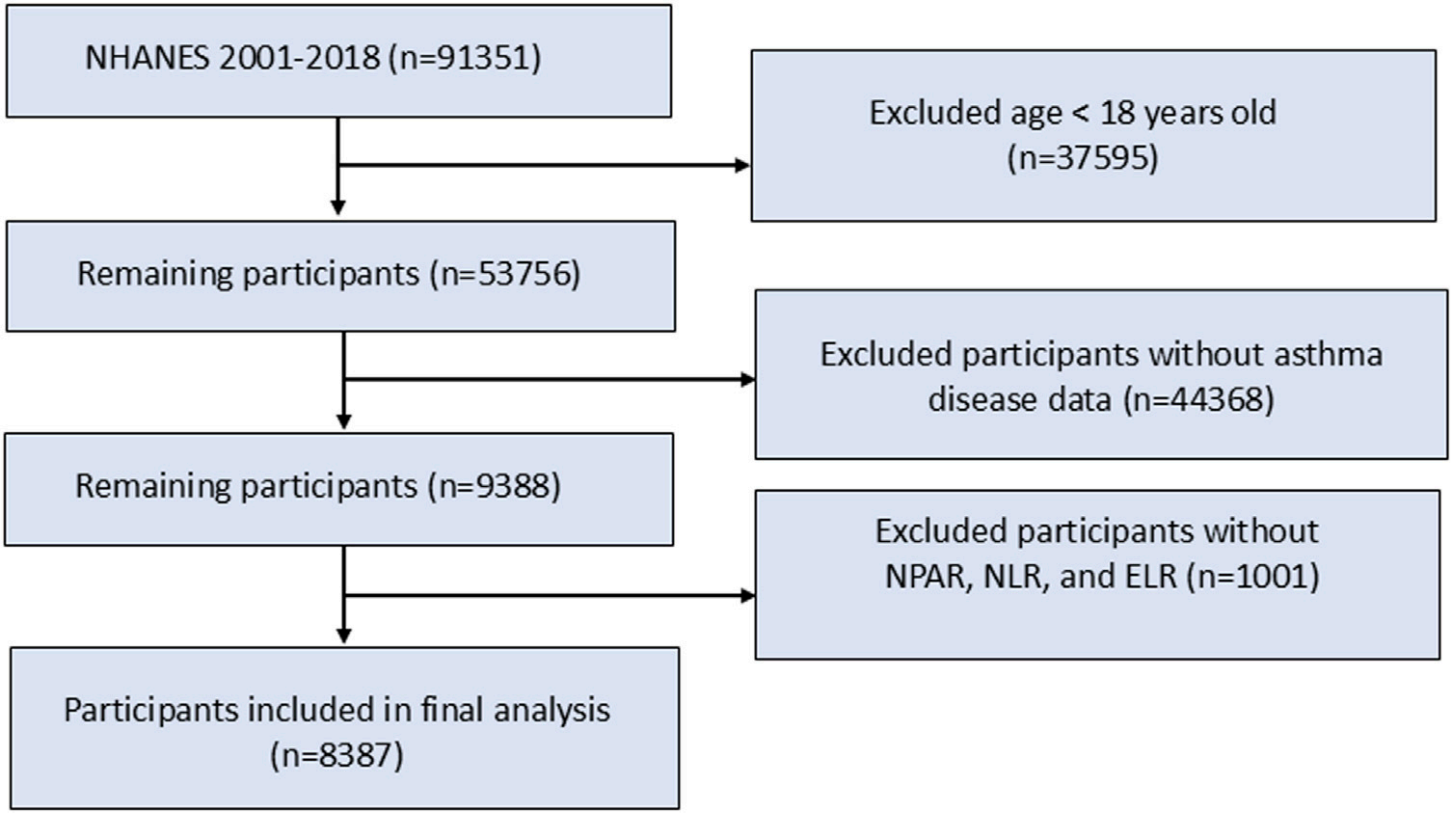
Flow chart of study population selection.

The study population consisted of adults aged 18 years and older who had a diagnosis of CRDs. Initially, a total of 91,351 participants were included in the NHANES survey cycles from 2001 to 2018. However, after excluding participants without complete data on CRDs status, hematologic inflammatory biomarkers (NPAR, NLR, ELR), and other covariates, a final sample of 8,387 CRDs patients was included in the analysis (Figure 1).

### Assessment of mortality

All-cause mortality data were obtained by linking the NHANES dataset to the National Death Index (NDI) as of 26 April 2022. The NDI provides information on the date and cause of death for individuals in the NHANES dataset.

### Statistical analysis

Multivariable weighted Cox regression analysis was used to assess the association between the inflammatory biomarkers (NPAR, NLR, ELR) and all-cause mortality. The current observational study utilized three models following the guidelines of Strengthening the Reporting of Observational Studies in Epidemiology (STROBE) (von Elm et al., 2007). Model 1 did not include any adjustments. Model 2 was adjusted for age, sex, and race, as these demographic factors are known to influence both asthma outcomes and mortality risk. Model 3 included all variables from Model 2, plus additional confounders such as marital status, education level, body mass index (BMI), and smoking status. To explore the non-linear relationship between the inflammatory biomarkers and mortality, smoothing curve fitting was performed. Kaplan-Meier survival curves were generated to visualize survival differences between groups stratified by the levels of inflammatory biomarkers. Additionally, receiver operating characteristic (ROC) curve analysis was conducted to evaluate the diagnostic performance of the inflammatory biomarkers in predicting mortality, with the area under the ROC curve (AUC) used to compare the predictive accuracy of the biomarkers.

## Results

### Baseline characteristics of the study population

A total of 8,387 adults with chronic respiratory diseases (CRDs) were included in the analysis. The mean age of the participants was 47.9 years, with a standard deviation (SD) of 19.4 years. The majority of the participants were female (57.1%) and non-Hispanic White (50.3%). The baseline demographic and clinical characteristics of the study population are summarized in Table 1. Compared to those assumed alive, participants who were assumed deceased were significantly older (mean age 68.4 years vs. 44.1 years, P < 0.001), more likely to be male (51.5% vs. 41.4%, P < 0.001), and had a higher proportion of non-Hispanic White individuals (67.1% vs. 47.2%, P < 0.001). Additionally, deceased participants had higher levels of NPAR (mean 154.6 vs. 139.8, P < 0.001), NLR (mean 2.9 vs. 2.2, P < 0.001), and neutrophils (mean 4.8 vs. 4.4, P < 0.001), while having lower levels of albumin (mean 40.4 vs. 41.9, P < 0.001) and lymphocytes (mean 2.0 vs. 2.2, P < 0.001).

**TABLE 1:** Demographic and clinical characteristics according to mortality status.

Characteristics	Total	All-cause mortality		P-value
		Assumed alive	Assumed deceased	
Age (years), Mean ± SD	47.9 ± 19.4	44.1 ± 18.0	68.4 ± 13.5	<0.001
Sex, n (%)				<0.001
Male	3,596 (42.9)	2,923 (41.4)	673 (51.5)	
Female	4,778 (57.1)	4,143 (58.6)	635 (48.5)	
Race, n (%)				<0.001
Mexican	862 (10.3)	775 (11)	87 (6.7)	
Hispanics	721 (8.6)	660 (9.3)	61 (4.7)	
Non-Hispanic White	4,216 (50.3)	3,338 (47.2)	878 (67.1)	
Non-Hispanic Black	1894 (22.6)	1,662 (23.5)	232 (17.7)	
Others	681 (8.1)	631 (8.9)	50 (3.8)	
Education levels, n (%)				<0.001
≤High school	3,731 (44.6)	2,922 (41.4)	809 (61.9)	
College	2,571 (30.7)	2,236 (31.6)	335 (25.6)	
> College	1,514 (18.1)	1,358 (19.2)	156 (11.9)	
Others	558 (6.7)	550 (7.8)	8 (0.6)	
Marital status, n (%)				<0.001
Married or living with a partner	4,232 (50.5)	3,622 (51.3)	610 (46.6)	
Not married	3,822 (45.6)	3,126 (44.2)	696 (53.2)	
Others	320 (3.8)	318 (4.5)	2 (0.2)	
BMI (kg/m2), Mean ± SD	30.2 ± 7.9	30.3 ± 8.0	29.3 ± 7.6	<0.001
Smoking status, n (%)				<0.001
Yes	4,324 (54.2)	3,365 (50.4)	959 (73.7)	
No	3,651 (45.8)	3,309 (49.6)	342 (26.3)	
Others	4 (0.0)	3 (0)	1 (0.1)	
NPAR, Mean ± SD	142.1 ± 31.1	139.8 ± 29.7	154.6 ± 35.3	<0.001
NLR, Mean ± SD	2.3 ± 1.4	2.2 ± 1.2	2.9 ± 2.0	<0.001
ELR, Median (IQR)	0.1 (0.1, 0.1)	0.1 (0.1, 0.1)	0.1 (0.1, 0.2)	<0.001
ALBUMIN, Mean ± SD	41.7 ± 3.8	41.9 ± 3.7	40.4 ± 3.9	<0.001
Lymphocyte, Mean ± SD	2.2 ± 1.4	2.2 ± 1.3	2.0 ± 1.8	<0.001
Eosinophil, Median (IQR)	0.2 (0.1, 0.3)	0.2 (0.1, 0.3)	0.2 (0.1, 0.3)	0.004
Neutrophil, Mean ± SD	4.5 ± 1.9	4.4 ± 1.9	4.8 ± 1.9	<0.001

Mean ± standard deviation (SD) for continuous variables, percentages (%) for categorical variables. BMI, body mass index; NPAR, neutrophil percentage-to-albumin ratio; NLR, neutrophil-to-lymphocyte ratio; ELR, eosinophil-to-lymphocyte ratio.

### Association between inflammatory biomarkers and all-cause mortality

Multivariable Cox regression analysis was performed to assess the association between inflammatory biomarkers and all-cause mortality in adults with CRDs. The results are presented in Table 2. In the fully adjusted model (Model 3), higher levels of NPAR were significantly associated with increased all-cause mortality (HR = 1.33, 95% CI: 1.26–1.40, P < 0.001). Similarly, elevated NLR was associated with higher mortality risk (HR = 1.24, 95% CI: 1.17–1.30, P < 0.001). In contrast, ELR did not show a significant association with mortality (HR = 1.04, 95% CI: 0.99–1.10, P = 0.098). The hazard ratios for each quartile of NPAR and NLR are also shown in Table 2, indicating a dose-response relationship between these biomarkers and mortality risk. To further investigate the predictive performance of these biomarkers within specific disease contexts, we conducted a subgroup analysis on the three major CRD subtypes in our cohort: asthma, emphysema, and chronic bronchitis. The results of these analyses, which are presented in Supplementary Table 1 and Supplementary Table 2, demonstrated that higher levels of NPAR and NLR were consistently associated with an increased risk of all-cause mortality across all three conditions in all three models.

**TABLE 2:** Multivariable analysis on the associations between NPAR, NLR, ELR, albumin lymphocyte, eosinophil, neutrophil and all-cause mortality.

Biomarker	Model 1		Model 2		Model 3	
	HR (95% CI)	P-value	HR (95% CI)	P-value	HR (95% CI)	P-value
NAPR	1.51 (1.43∼1.59)	<0.001	1.34 (1.27∼1.42)	<0.001	1.33 (1.26∼1.4)	<0.001
Q1	1(Ref)		1(Ref)		1(Ref)	
Q2	1.45 (1.2∼1.76)	<0.001	1.25 (1.03∼1.52)	0.021	1.21 (1∼1.47)	0.049
Q3	1.88 (1.57∼2.26)	<0.001	1.39 (1.16∼1.68)	<0.001	1.36 (1.13∼1.64)	0.001
Q4	3.41 (2.88∼4.03)	<0.001	2.37 (2∼2.82)	<0.001	2.28 (1.92∼2.71)	<0.001
NLR	1.44 (1.37∼1.52)	<0.001	1.25 (1.19∼1.32)	<0.001	1.24 (1.17∼1.3)	<0.001
Q1	1(Ref)		1(Ref)		1(Ref)	
Q2	1.2 (1∼1.45)	0.051	1.1 (0.92∼1.33)	0.301	1.12 (0.93∼1.35)	0.235
Q3	1.4 (1.17∼1.67)	<0.001	1.22 (1.02∼1.46)	0.031	1.2 (1.01∼1.44)	0.044
Q4	2.84 (2.42∼3.33)	<0.001	1.89 (1.6∼2.23)	<0.001	1.83 (1.55∼2.17)	<0.001
ELR	1.24 (1.18∼1.3)	<0.001	1.04 (0.99∼1.1)	0.098	1.04 (0.99∼1.1)	0.098
Q1	1(Ref)		1(Ref)		1(Ref)	
Q2	1.07 (0.9∼1.27)	0.441	0.89 (0.75∼1.06)	0.192	0.89 (0.75∼1.06)	0.192
Q3	1.26 (1.07∼1.49)	0.006	0.96 (0.81∼1.13)	0.602	0.96 (0.81∼1.13)	0.602
Q4	1.84 (1.58∼2.15)	<0.001	1.09 (0.93∼1.27)	0.301	1.09 (0.93∼1.27)	0.301
Albumin	0.72 (0.68∼0.75)	<0.001	0.76 (0.72∼0.8)	<0.001	0.76 (0.72∼0.8)	<0.001
Q1	1(Ref)		1(Ref)		1(Ref)	
Q2	0.7 (0.61∼0.81)	<0.001	0.58 (0.5∼0.66)	<0.001	0.56 (0.49∼0.65)	<0.001
Q3	0.53 (0.45∼0.62)	<0.001	0.48 (0.41∼0.56)	<0.001	0.48 (0.41∼0.57)	<0.001
Q4	0.36 (0.31∼0.42)	<0.001	0.43 (0.36∼0.5)	<0.001	0.42 (0.36∼0.5)	<0.001
Lymphocyte	0.72 (0.68∼0.75)	<0.001	0.93 (0.89∼0.98)	0.009	0.92 (0.87∼0.97)	0.002
Q1	1(Ref)		1(Ref)		1(Ref)	
Q2	0.48 (0.42∼0.55)	<0.001	0.76 (0.66∼0.87)	<0.001	0.76 (0.66∼0.87)	<0.001
Q3	0.36 (0.31∼0.43)	<0.001	0.73 (0.62∼0.85)	<0.001	0.71 (0.6∼0.83)	<0.001
Q4	0.4 (0.34∼0.46)	<0.001	0.84 (0.72∼0.99)	0.032	0.81 (0.69∼0.95)	0.008
Eosinophil	1.06 (1∼1.13)	0.053	1 (0.94∼1.06)	0.893	1 (0.94∼1.06)	0.893
Q1	1(Ref)		1(Ref)		1(Ref)	
Q2	0.54 (0.42∼0.69)	<0.001	0.61 (0.47∼0.78)	<0.001	0.61 (0.47∼0.78)	<0.001
Q3	0.66 (0.51∼0.85)	0.001	0.63 (0.49∼0.81)	<0.001	0.63 (0.49∼0.81)	<0.001
Q4	0.7 (0.55∼0.9)	0.006	0.68 (0.53∼0.87)	0.003	0.68 (0.53∼0.87)	0.003
Neutrophil	1.2 (1.14∼1.26)	<0.001	1.26 (1.19∼1.33)	<0.001	1.22 (1.15∼1.28)	<0.001
Q1	1(Ref)		1(Ref)		1(Ref)	
Q2	1.39 (1.17∼1.66)	<0.001	1.15 (0.97∼1.38)	0.112	1.16 (0.97∼1.38)	0.109
Q3	1.58 (1.34∼1.87)	<0.001	1.38 (1.16∼1.64)	<0.001	1.36 (1.14∼1.62)	0.001
Q4	1.8 (1.53∼2.13)	<0.001	1.96 (1.66∼2.33)	<0.001	1.79 (1.5∼2.12)	<0.001

Abbreviations: NPAR, neutrophil percentage-to-albumin ratio; NLR, neutrophil-to-lymphocyte ratio; ELR, eosinophil-to-lymphocyte ratio; HR, hazard ratio; CI, confidence interval.

Model 1: adjust for none.

Model 2: adjust for age, sex, and race.

Model 3: adjust for age, sex, race, marital status, education level, body mass index, smoking status.

**FIGURE 2:**
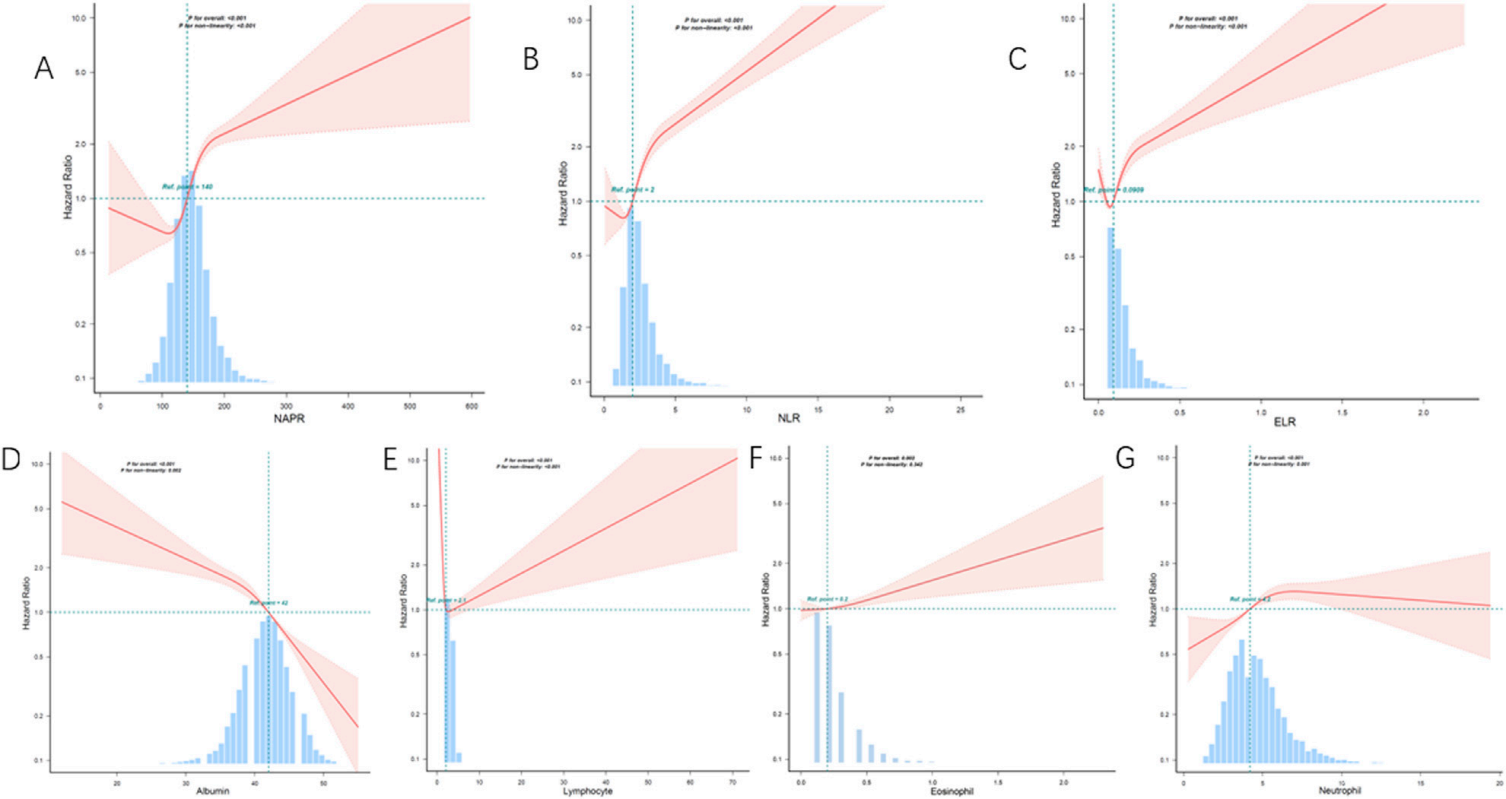
Restricted cubic spline analysis of the association between inflammatory biomarkers and all-cause mortality. **(A)** NAPR, **(B)** NLR, **(C)** ELR, **(D)** Albumin, **(E)** Lymphocyte count, **(F)** Eosinophil count, **(G)** Neutrophil count. The solid lines represent the hazard ratios, and the shaded areas indicate the 95% confidence intervals. The histograms show the distribution of the data for each biomarker.

**FIGURE 3:**
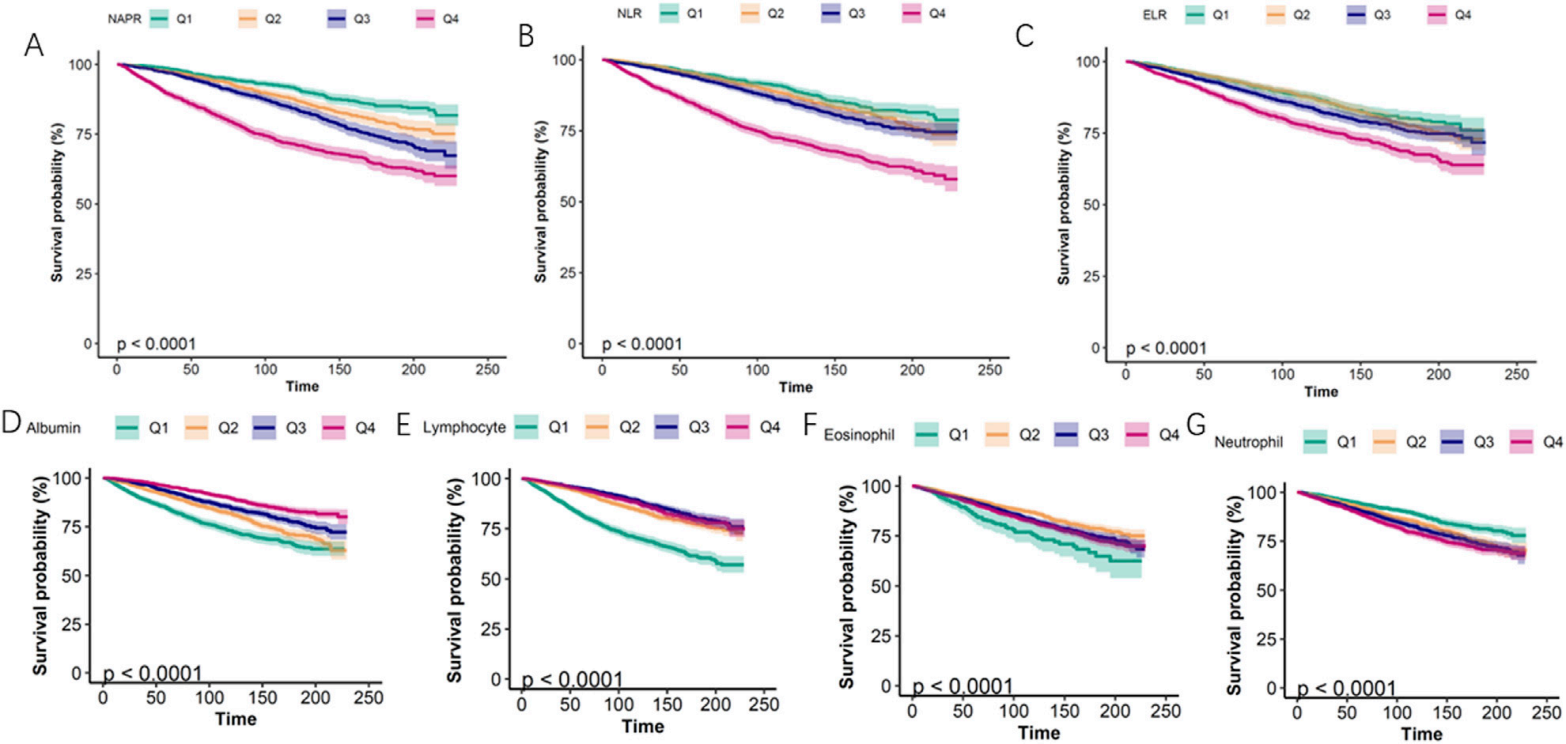
Kaplan–Meier survival curves for all-cause mortality stratified by quartiles (Q1-Q4) of inflammatory biomarkers. **(A)** NAPR, **(B)** NLR, **(C)** ELR, **(D)** Albumin, **(E)** Lymphocyte count, **(F)** Eosinophil count, **(G)** Neutrophil count.

Smoothing curve fitting analysis indicated a non-linear correlation between the inflammatory biomarkers (NPAR, NLR, ELR, albumin, eosinophil, lymphocyte, and neutrophil counts) and mortality (Figure 2). This suggests that the relationship between these biomarkers and mortality risk may not be strictly linear, highlighting the complexity of inflammatory responses in asthma patients. Kaplan-Meier survival curves further illustrated that patients with higher levels of NPAR, NLR, and neutrophil counts experienced higher all-cause mortality rates. Conversely, individuals with elevated levels of albumin, eosinophils, and lymphocyte counts had lower mortality rates (Figure 3).

**FIGURE 4:**
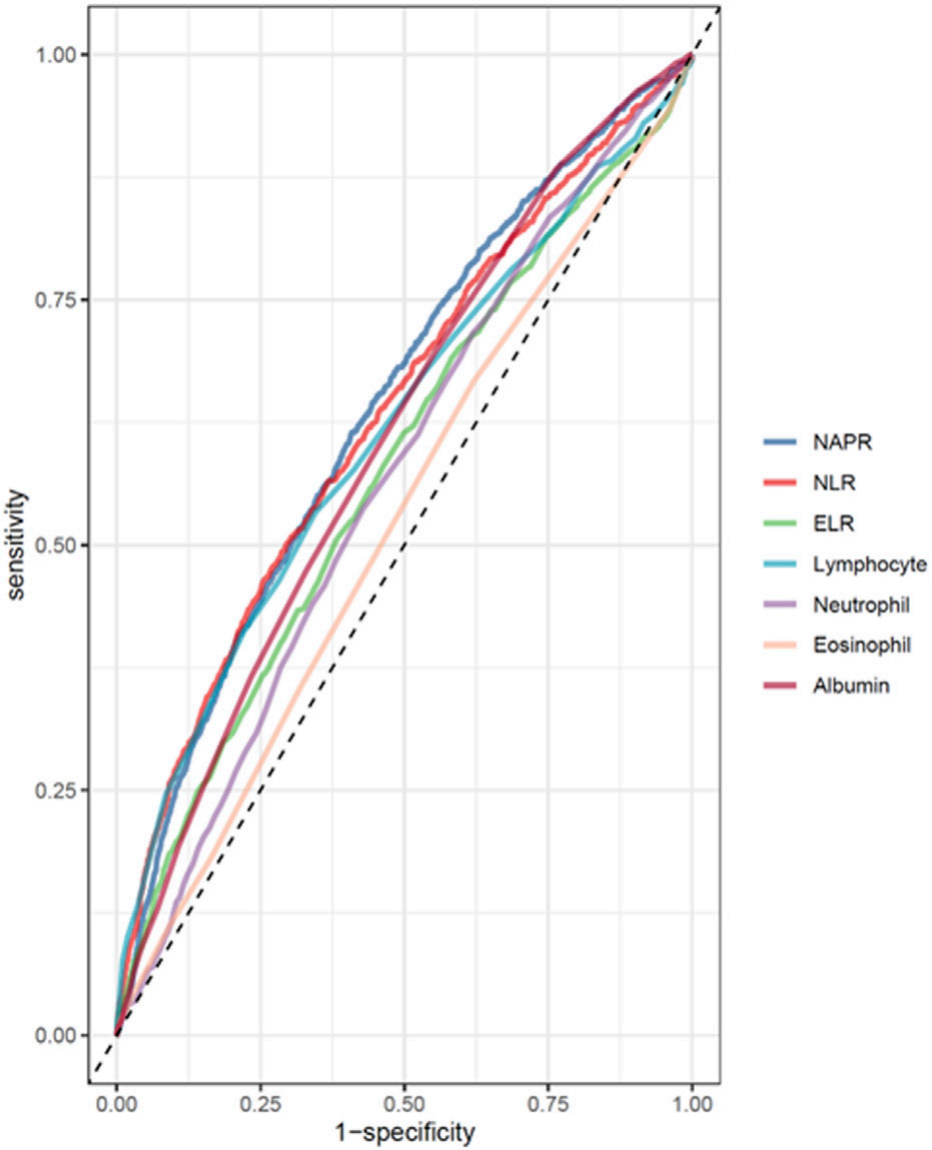
ROC curves and the AUC values of the inflammatory markers (NPAR, NLR, ELR, albumin, eosinophil, lymphocyte, and neutrophil counts).

ROC curve analysis (Table 3) revealed modest predictive performance for NPAR (AUC = 0.639, 95% CI: 0.623–0.656) and NLR (AUC = 0.634, 95% CI: 0.617–0.650), with ELR showing lower performance (AUC = 0.578, 95% CI: 0.560–0.596). These findings support NPAR and NLR as complementary risk indicators that may enhance mortality prediction when integrated with established clinical parameters, despite their modest individual discriminative power (AUC < 0.65) (Figure 4).

**TABLE 3:** Comparison of AUC values between the indicators of inflammation.

Biomarker	AUC	95% CI (lower)	95% CI (upper)	Best threshold	Specificity	Sensitivity
NPAR	0.639477	0.622989	0.655966	144.2164	0.5916	0.6147
NLR	0.633819	0.61667	0.650968	2.5963	0.745	0.4633
ELR	0.578021	0.560473	0.59557	0.1076	0.6192	0.5038
Albumin	0.607485	0.591228	0.623743	41.5	0.5611	0.5849
Lymphocyte	0.616117	0.598266	0.633969	1.65	0.7795	0.4113
Eosinophil	0.523943	0.507279	0.540608	0.15	0.3798	0.6667
Neutrophil	0.565904	0.549272	0.582537	4.45	0.572	0.5367

Abbreviations: NPAR, neutrophil percentage-to-albumin ratio; NLR, neutrophil-to-lymphocyte ratio; ELR, eosinophil-to-lymphocyte ratio; AUC, area under the curve; HR, hazard ratio; CI, confidence interval.

## Discussion

CRDs are characterized by chronic inflammation, which plays a central role in disease progression and mortality. In this study, we evaluated the predictive roles of three inflammatory biomarkers—NPAR, NLR, and ELR-in relation to mortality among adults with CRDs. Our findings revealed that higher levels of NPAR and NLR were significantly associated with increased mortality risk, while ELR did not show a significant association. Our results align with studies beyond CRDs, where hematologic biomarkers guide clinical decisions. In pneumonia, CRP/MPV and NLR outperform standalone markers in predicting hospitalization needs, directly impacting antimicrobial strategies and resource allocation (Yeşildağ et al., 2024). This reinforces the translational value of ratio-based biomarkers in acute and chronic settings. Specifically in COPD, elevated NLR signifies neutrophilic inflammation and predicts mortality independent of traditional factors (Paliogiannis et al., 2018), while in asthma, NPAR recently emerged as a cross-sectional predictor of severity (Bi et al., 2025).

Our study found that higher NPAR levels were associated with increased mortality risk in adults with CRDs (HR = 1.33, 95% CI: 1.26–1.40, P < 0.001). This result aligns with existing literature that underscores the predictive value of NPAR in various inflammatory conditions, including cardiovascular diseases and cancers. NPAR is a composite biomarker that integrates the percentage of neutrophils in the total white blood cell count with serum albumin levels, reflecting both the inflammatory response and nutritional status (Bi et al., 2025). The association between elevated NPAR and increased mortality risk has been documented in multiple studies. For instance, a systematic review highlighted that low serum albumin levels and high neutrophil percentages are indicative of poor prognosis in cancer patients (Gupta and Lis, 2010). Additionally, researcher demonstrated the diagnostic significance of NPAR in patients with infectious meningitis, reinforcing its role as a comprehensive indicator of systemic inflammation (Bughio et al., 2024). Furthermore, Cui et al. reported that NPAR serves as an independent predictor of in-hospital mortality in patients with ST-segment elevation myocardial infarction, illustrating its utility in acute clinical settings (Cui et al., 2019). Similarly, Peng et al. found a strong correlation between elevated NPAR levels and mortality in patients with cardiogenic shock, further supporting the biomarker’s prognostic capabilities (Peng et al., 2020). The findings from these studies collectively suggest that NPAR can be a valuable tool for risk stratification in patients with CRDs, as it encapsulates critical aspects of both inflammatory response and nutritional status.

NLR, which measures the ratio of neutrophils to lymphocytes, is a widely studied biomarker that reflects the balance between pro-inflammatory and anti-inflammatory immune responses. Elevated NLR has been associated with poor outcomes in several inflammatory conditions, including COPD and asthma. In our study, higher NLR levels were significantly associated with increased mortality risk (HR = 1.24, 95% CI: 1.17–1.30, P < 0.001). This finding underscores the importance of systemic inflammation in the pathophysiology of CRDs and highlights the potential utility of NLR as a prognostic marker in this population.

NLR is a biomarker that quantifies the relative proportions of neutrophils and lymphocytes in the bloodstream, reflecting the balance between pro-inflammatory and anti-inflammatory immune responses. This ratio has garnered significant attention in clinical research due to its ability to serve as an indicator of systemic inflammation. Elevated NLR levels have been associated with poor clinical outcomes across various inflammatory conditions, including chronic obstructive pulmonary disease (COPD) and asthma (Pascual-González et al., 2018; Furutate et al., 2016). In our study, we found that higher NLR levels were significantly associated with increased mortality risk among adults with CRDs, with a HR of 1.24 (95% CI: 1.17–1.30, P < 0.001). This finding underscores the critical role of systemic inflammation in the pathophysiology of CRDs and highlights the potential utility of NLR as a prognostic marker in this population. The underlying mechanisms for the prognostic significance of NLR in CRDs can be attributed to the role of neutrophils in the inflammatory process. Neutrophils are among the first responders to inflammation and are involved in the pathogenesis of lung diseases by releasing reactive oxygen species and proteolytic enzymes, which can lead to tissue damage and exacerbation of respiratory conditions (Xiong et al., 2017). In contrast, lymphocytes play a crucial role in the anti-inflammatory response. An imbalance favoring neutrophils over lymphocytes, as indicated by a higher NLR, suggests a state of chronic inflammation that may contribute to the progression of CRDs and associated complications (Paliogiannis et al., 2018). Moreover, the NLR has been shown to correlate with other inflammatory markers, such as interleukin-6 (IL-6) and C-reactive protein (CRP), which further supports its role as a comprehensive indicator of systemic inflammation (Zinellu et al., 2022; Lee et al., 2016). The ability of NLR to predict outcomes in various clinical settings, including acute exacerbations of COPD and other inflammatory diseases, reinforces its potential as a valuable tool for risk stratification and management in patients with CRDs (Sakurai et al., 2018).

ELR is a biomarker that evaluates the relative proportions of eosinophils and lymphocytes in the bloodstream. However, in our study, we did not find a significant association between ELR and mortality among patients with CRDs, with a HR of 1.04 (95% CI: 0.99–1.10, P = 0.098). This finding suggests that ELR may not be as effective as other inflammatory biomarkers, such as the NPAR or the NLR, in predicting mortality in this population. The lack of a significant association between ELR and mortality could be attributed to the diverse inflammatory profiles present in CRDs.

NPAR showed modest prognostic utility (AUC 0.639) in discriminating mortality risk, outperforming other biomarkers in this cohort though still within a limited predictive range. Its clinical value lies primarily as an accessible adjunct to comprehensive risk assessment tools. These findings suggest that NPAR may be a more reliable predictor of mortality in patients with CRDs compared to NLR, ELR, albumin, lymphocyte, and eosinophil counts. The identification of reliable biomarkers for risk stratification and prognosis is crucial for improving patient outcomes in CRDs. NPAR and NLR, being easily measurable from routine blood tests, offer a practical and cost-effective approach for identifying high-risk patients. These biomarkers could potentially guide clinical decision-making, informing personalized treatment strategies and optimizing healthcare resource allocation.

We acknowledge several limitations in our study. First, the cross-sectional design of the NHANES data limits our ability to establish causality, and prospective cohort studies are needed to confirm the temporal relationship between the biomarkers and mortality. Second, although we adjusted for multiple covariates, the possibility of residual confounding cannot be completely excluded. Third, the study population was limited to the United States, which may affect the generalizability of our findings to other regions. Additionally, the inflammatory biomarkers were measured at a single time point, which may not fully capture the dynamic nature of inflammation in CRDs. Future studies should incorporate repeated measurements of these biomarkers over time to better understand their predictive value and the natural history of CRDs. Furthermore, we recognize that our multivariable Cox regression models did not account for time-varying covariates or competing risks, which could have an impact on our results. We also acknowledge that our ROC-based approach for analyzing survival data may be suboptimal, as its primary purpose was to provide a straightforward comparison of the relative predictive capabilities of these biomarkers at a single time point. Future research could explore more appropriate methods for survival data, such as time-dependent ROC curve analysis, for a more comprehensive evaluation of predictive performance. Finally, while NHANES is a robust dataset, potential misclassification bias in self-reported CRD status or other variables cannot be entirely excluded.”

In conclusion, our study identifies NPAR and NLR as potential prognostic biomarkers for all-cause mortality in adults with chronic respiratory diseases, with NPAR showing a slight edge in predictive ability. We acknowledge that the modest AUC values and the limitations of our study design mean these biomarkers are not yet ready for independent clinical use. Instead, our findings suggest NPAR and NLR could serve as routine availability and low cost support their role as practical complementary biomarkers for initial risk stratification in CRD management. We call for future prospective studies to validate these findings and establish definitive clinical cutoffs, thereby paving the way for their potential application in patient care.

## Data Availability

The original contributions presented in the study are included in the article/Supplementary Material, further inquiries can be directed to the corresponding author.
