# Supplementary material for: NHANES-based assessment of neutrophil-percentage-to-albumin and neutrophil-to-lymphocyte ratios as moderate predictors of mortality in adults with chronic respiratory diseases
Source: Front Pharmacol. 2025 Sep 26;16:1582120. doi: 10.3389/fphar.2025.1582120 (PMC12510946; doi:10.3389/fphar.2025.1582120)
Supplement: Supplementary file 1 [file Table1.docx]

**Supplemantary Table 1**. Association between NPAR and all-cause mortality in patients with asthma, emphysema, and chronic bronchitis.

|  | **Model 1** | | **Model 2** | | **Model 3** | |
| --- | --- | --- | --- | --- | --- | --- |
| **Variable** | **HR (95% CI)** | **P-value** | **HR (95% CI)** | **P-value** | **HR (95% CI)** | **P-value** |
| **Asthma** | **1.47 (1.37~1.57)** | **<0.001** | **1.34 (1.25~1.43)** | **<0.001** | **1.29 (1.21~1.39)** | **<0.001** |
| Q1 | Reference |  | Reference |  | Reference |  |
| Q2 | 1.46 (1.14~1.86) | 0.002 | 1.28 (1.01~1.64) | 0.045 | 1.26 (0.98~1.61) | 0.072 |
| Q3 | 1.77 (1.4~2.25) | <0.001 | 1.43 (1.13~1.81) | 0.003 | 1.36 (1.06~1.74) | 0.014 |
| Q4 | 3.17 (2.55~3.94) | <0.001 | 2.37 (1.9~2.96) | <0.001 | 2.17 (1.72~2.72) | <0.001 |
| **Emphysema** | **1.67 (1.53~1.83)** | **<0.001** | **1.26 (1.15~1.39)** | **<0.001** | **1.22 (1.11~1.35)** | **<0.001** |
| Q1 | Reference |  | Reference |  | Reference |  |
| Q2 | 2.26 (1.61~3.16) | <0.001 | 1.11 (0.79~1.57) | 0.548 | 1.01 (0.71~1.44) | 0.964 |
| Q3 | 3.03 (2.19~4.2) | <0.001 | 1.19 (0.85~1.66) | 0.319 | 1.09 (0.77~1.54) | 0.635 |
| Q4 | 5.4 (3.94~7.39) | <0.001 | 1.88 (1.36~2.61) | <0.001 | 1.68 (1.2~2.34) | 0.002 |
| **Chronic bronchitis** | **1.57 (1.45~1.7)** | **<0.001** | **1.42 (1.31~1.54)** | **<0.001** | **1.36 (1.25~1.48)** | **<0.001** |
| Q1 | Reference |  | Reference |  | Reference |  |
| Q2 | 1.52 (1.14~2.03) | 0.005 | 1.27 (0.94~1.7) | 0.115 | 1.17 (0.86~1.58) | 0.323 |
| Q3 | 2.03 (1.54~2.68) | <0.001 | 1.57 (1.19~2.09) | 0.002 | 1.51 (1.12~2.02) | 0.006 |
| Q4 | 3.83 (2.96~4.97) | <0.001 | 2.72 (2.09~3.55) | <0.001 | 2.35 (1.78~3.11) | <0.001 |

**Abbreviations**: NPAR, neutrophil percentage-to-albumin ratio.

Model 1: adjust for none.

Model 2: adjust for age, sex, and race.

Model 3: adjust for age, sex, race, marital status, education level, body mass index, smoking status.

**Supplemantary Table 2**. Association between NLR and all-cause mortality in patients with asthma, emphysema, and chronic bronchitis.

|  | **Model 1** | | **Model 2** | | **Model 3** | |
| --- | --- | --- | --- | --- | --- | --- |
| **Variable** | **HR (95% CI)** | **P-value** | **HR (95% CI)** | **P-value** | **HR (95% CI)** | **P-value** |
| **Asthma** | **1.39 (1.3~1.48)** | **<0.001** | **1.26 (1.18~1.35)** | **<0.001** | **1.25 (1.17~1.34)** | **<0.001** |
| Q1 | Reference |  | Reference |  | Reference |  |
| Q2 | 1.32 (1.05~1.68) | 0.02 | 1.15 (0.9~1.45) | 0.264 | 1.15 (0.91~1.46) | 0.252 |
| Q3 | 1.45 (1.15~1.83) | 0.002 | 1.37 (1.08~1.73) | 0.01 | 1.36 (1.07~1.72) | 0.012 |
| Q4 | 2.67 (2.17~3.3) | <0.001 | 1.95 (1.57~2.43) | <0.001 | 1.89 (1.52~2.36) | <0.001 |
| **Emphysema** | **1.59 (1.46~1.74)** | **<0.001** | **1.19 (1.09~1.3)** | **<0.001** | **1.18 (1.07~1.3)** | **0.001** |
| Q1 | Reference |  | Reference |  | Reference |  |
| Q2 | 1.67 (1.21~2.3) | 0.002 | 1.12 (0.8~1.55) | 0.518 | 1.1 (0.78~1.54) | 0.596 |
| Q3 | 2.23 (1.64~3.02) | <0.001 | 1.15 (0.84~1.58) | 0.391 | 1.09 (0.78~1.52) | 0.612 |
| Q4 | 4.14 (3.1~5.52) | <0.001 | 1.64 (1.22~2.22) | 0.001 | 1.6 (1.18~2.19) | 0.003 |
| **Chronic bronchitis** | **1.5 (1.39~1.62)** | **<0.001** | **1.3 (1.2~1.41)** | **<0.001** | **1.23 (1.14~1.34)** | **<0.001** |
| Q1 | Reference |  | Reference |  | Reference |  |
| Q2 | 1.31 (0.99~1.73) | 0.055 | 1.31 (0.99~1.74) | 0.06 | 1.22 (0.91~1.63) | 0.176 |
| Q3 | 1.38 (1.05~1.82) | 0.02 | 1.28 (0.97~1.7) | 0.081 | 1.22 (0.92~1.64) | 0.171 |
| Q4 | 3.24 (2.54~4.13) | <0.001 | 2.24 (1.74~2.88) | <0.001 | 1.88 (1.45~2.45) | <0.001 |

**Abbreviations**: NLR, neutrophil-to-lymphocyte ratio.

Model 1: adjust for none.

Model 2: adjust for age, sex, and race.

Model 3: adjust for age, sex, race, marital status, education level, body mass index, smoking status.
